# Evaluation of poly(amidoamine) dendrimers as potential carriers of iminodiacetic derivatives using solubility studies and 2D-NOESY NMR spectroscopy

**DOI:** 10.1007/s10867-012-9277-5

**Published:** 2012-08-16

**Authors:** Magdalena Markowicz, Paweł Szymański, Marcin Ciszewski, Arkadiusz Kłys, Elżbieta Mikiciuk-Olasik

**Affiliations:** 1 Department of Pharmaceutical Chemistry and Drug Analysis, Medical University of Lodz, Muszynskiego 1, 90-151 Lodz, Poland; 2 Laboratory of Molecular Spectroscopy, Department of Chemistry, University of Lodz, Tamka 12, 91-403 Lodz, Poland

**Keywords:** PAMAM dendrimers, Iminodiacetic acid, Solubility studies, Electrostatic interactions, MRI contrast agents

## Abstract

The interactions between dendrimers and different types of drugs are nowadays one of the most actively investigated areas of the pharmaceutical sciences. The interactions between dendrimers and drugs can be divided into: internal encapsulation, external electrostatic interaction, and covalent conjugation. In the present study, we investigated the potential of poly(amidoamine) (PAMAM) dendrimers for solubility of four iminodiacetic acid derivatives. We reported that PAMAM dendrimers contribute to significant solubility enhancement of iminodiacetic acid analogues. The nature of the dendrimer–drug complexes was investigated by ^1^H NMR and 2D-NOESY spectroscopy. The ^1^H NMR analysis proved that the water-soluble supramolecular structure of the complex was formed on the basis of ionic interactions between terminal amine groups of dendrimers and carboxyl groups of drug molecules, as well as internal encapsulation. The 2D-NOESY analysis revealed interactions between the primary amine groups of PAMAM dendrimers and the analogues of iminodiacetic acid. The results of solubility studies together with ^1^H NMR and 2D-NOESY experiments suggest that the interactions between PAMAM dendrimers of generation 1–4 and derivatives of iminodiacetic acid are based on electrostatic interactions and internal encapsulation.

**Electronic supplementary material** The online version of this article (doi:10.1007/s10867-012-9277-5) contains supplementary material, which is available to authorized users.

## Introduction

Dendrimers, a relatively new class of chemical compounds, are large, complex molecules, which possess, in comparison to traditional linear polymers, well-defined chemical structure. Dendrimers are multi-branched, three-dimensional polymers with low polydispersity and high functionality [1]. A typical dendrimer is composed of three elements: an initiator core, interior layers known as generations that are built of repeating units, attached to the initiator core, and multiple peripheral functional groups that are attached to the outermost interior generation [1–5]. Polyamidoamine (PAMAM) dendrimers were the first dendrimers that were synthesized [6, 7]. These molecules and their modifications have received widespread attention throughout the world and are under the most active investigation.

Over the past decade, interest in utilization of dendrimers as drug delivery systems has increased. PAMAM dendrimers, which are the most precisely examined dendrimer family, appear to be safe for potential use in a wide variety of therapeutic applications for human diseases [8]. The utilization of dendrimers as molecular containers was proposed for the first time by Maciejewski in 1982 [9]. Nowadays host-guest properties of dendritic polymers are under scientific investigation and have gained a crucial position in the field of supramolecular chemistry. Encapsulation of guest molecules into dendritic structures might be based on hydrogen bonding interaction. Fox and co-workers conducted research in which they showed that lower generation PAMAM dendrimers incorporate guest molecules at external (surface amino groups) and internal (interior amide groups) coordination sites [10, 11]. Interaction between biologically active compounds and dendrimers may also be a consequence of electrostatic interactions which may occur between PAMAM dendrimers and acidic, water insoluble molecules such as benzoic acid and salicylic acid. Non-polar groups at the ends of dendrimer branches enable dendrimers to act as micelles which may be utilized as molecular vehicles to transport guest molecules between organic and inorganic phases [3, 11].

There are numerous studies confirming that water soluble dendrimers are capable of binding and solubilizing small acidic molecules with low water solubility [12, 13]. PAMAM dendrimers with amine-terminated surface groups might be potential carriers for NSAIDs (non-steroidal anti-inflammatory drugs) which possess carboxyl groups. There are several NSAIDs which have been successfully encapsulated into or complexed with PAMAM (e.g., aspirin, indomethacin, flurbiprofen, ketoprofen, ibuprofen, diclofenac and naproxen) [14–16]. Apart from non-steroidal anti-inflammatory drugs, dendrimers may be conjugated with anticancer drugs, such as cisplatin, camptothecin, paclitaxel and doxorubicin [17]. There has been much research describing the possibility of enclosing within the dendrimer structure not only drug molecules, but also genetic materials, targeting agents, and dyes either by encapsulation, complexation, or conjugation.

Magnetic resonance imaging (MRI), based on the knowledge gained in the study of nuclear magnetic resonance (NMR), is a non-invasive way of monitoring the internal organs and tissues of humans. Nowadays, it is a widely used diagnostic tool, which allows visualizing detailed internal structure (three-dimensional images) and limited function of the body. MRI is a standard method of cancer diagnosis. Furthermore, it is also used in oncology to locate, stage, plan treatment and, potentially, find recurrence.

MRI contrast agents such as Magnevist (Gd(III)-diethylenetriaminepentaacetic acid (Gd(III)-DTPA)) or Dotaren (Gd(III)-*N*,*N*′,*N* ^″^,*N* ^″′^-tetracarboxymethyl-1,4,7,10-tetraazacyclododecane (Gd(III)-DOTA)), commercially available, are characterized by short circulation times and inefficient differentiation between normal and diseased tissues. Moreover, they do not target specific organs or regions of the body [18]. Improved contrast enhancement might be achieved by conjugation of gadolinium-based contrast agents with polymers such as poly(amino)acids, polysaccharides and proteins [19–22]. Nevertheless, better contrast properties of contrast agents attached to polymers are often associated with longer residence time in the body, which increases the risk of gadolinium ion toxicity. As a consequence, there have been attempts to conjugate gadolinium-based contrast agents with dendrimers. Such conjugates enable specific targeting and imaging of internal organs or tumors [23]. Mebrofenin, and the other three iminodiacetic acid analogues complexed with gadolinium, are contrasting compounds, which show high affinity to liver cells and enable high-resolution (MRI) imaging of this organ, which has become an important tool in a routine clinical liver imaging.

Approved for clinical use, gadolinium-based MRI contrast agents such as Gd-DTPA (gadopentetate dimeglumine, Magnevist, Schering AG) are not sufficiently specific and selective. Thus, in liver and biliary duct diseases, hepatotropic contrast agents enabling identification and differentiation of focal changes in liver and determination of reasons for cholestasia are still in demand [24]. Furthermore, iminodiacetic acid derivatives might be radiolabeled with technetium (^99m^Tc), and, as a result, form complexes such as ^99m^Tc-diosopropyl-IDA (DISIDA) and ^99m^Tc-bromotriethyl-IDA (mebrofenin), which are utilized in cholescintigraphy. Unlike some radiopharmaceuticals that have fallen into disuse with time or whose indications are severely reduced, ^99m^Tc-IDA imaging agents have shown remarkable staying power and, in fact, their clinical indications have increased over the years. The analogues of iminodiacetic acid (IDA) labeled with technetium-99m are used in nuclear hepatology for non-invasive and quantitative evaluation of numerous hepatobiliary diseases related to bile formation and excretion [25, 26].

During the past decade, dendrimers have proved to be promising candidates in the design of new drug delivery systems. Thus, it is of vital importance to develop the theme of utilization of iminodiacetic acid derivatives in MRI of the hepatobiliary tract. In this study, we examined the effect of PAMAM dendrimers on solubility of iminodiacetic acid derivatives. The interactions between dendrimers and iminodiacetic acid analogues were further characterized by ^1^H NMR and two-dimensional nuclear Overhauser effect spectroscopy (2D-NOESY). Development of noninvasive delivery systems of MRI contrast agents is still a burning question for effective diagnosis of hepatobiliary tract diseases and attracts increasing attention from scientists today. To the best of our knowledge, little information concerning interactions between dendrimers and drugs of different biological activity which possess two carboxylic groups is available. Furthermore, there is no reference devoted to the use of PAMAM dendrimers as drug carriers of iminodiacetic acid derivatives at current stage, and 2D-NOESY was firstly employed to study the interaction mechanisms between dendrimers and these drug molecules.

## Experimental methods

### Materials and synthesis of derivatives of *N*-(2-phenylamine- 2 oxoethyl)-iminodiacetic acid

PAMAM dendrimers generation 1–4 were purchased from Sigma-Aldrich (St. Louis, MO, USA). Substrates for synthesis of iminodiacetic acid derivatives such as nitrilotriacetic acid, 4-methylaniline, 2,4-dimethylaniline, 2,4,6-trimethylaniline and 3-bromo-2,4,6-trimethyloaniline were purchased from Sigma-Aldrich. Acetic anhydride was obtained from PHPU Eurochem BGD Sp z o.o. (Poland). Pyridine was bought from Sigma-Aldrich. Methyl alcohol, ethyl alcohol, dichloromethane, sodium hydroxide, and hydrochloric acid were purchased from POCh S.A (Poland). All the chemicals were used without further purification. In the aqueous solubility studies, double-distilled water was used.

**Scheme 1:**
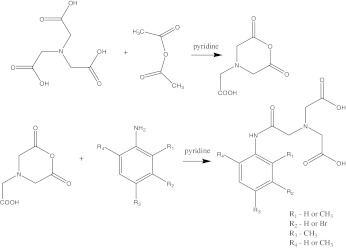
Synthesis of derivatives of *N*-(2-phenylamine- 2-oxoethyl)iminodiacetic acid

For synthesis of iminodiacetic acid derivatives, we applied method described by A. Nunn [27]. This method was previously used to synthesize *N*-(3-bromo-2,4,6-trimethylacetanilide)iminodiacetic acid (mebrofenin), but we also prepared other compounds by means of this method (Scheme 1).

Briefly, the first step of synthesis of *N*-(4-methylacetanilide)iminodiacetic acid leads to acquirement of nitrilotriacetic acid anhydride. In the second step in situ obtained anhydride reacts with 4-methylaniline in the environment of pyridine. The mixture was heated at 100°C for 2 h. After this time the mixture was cooled, the solvent was evaporated, and the residue was alkalized. It was then extracted with dichloromethane, followed by acidification with hydrochloric acid. The crude product was purified thanks to the crystallization from a mixture of ethanol and water. *N*-(4-methylacetanilide)iminodiacetic acid was synthesized and described for the first time by Burns et al. [28].

*N*-(2,4-dimethylacetanilide)iminodiacetic acid and *N*-(2,4,6-trimethylacetanilide)iminodiacetic acid were synthesized by the same procedure using 2,4-dimethylaniline and 2,4,6-trimethylaniline as substrates, respectively.

The structure of these compounds was confirmed by ^1^H NMR, ^13^C NMR, elemental analysis and IR spectra. Melting points were estimated by means of an electrothermal apparatus. A Mattson Infinity Series FT-IR spectrophotometer was used to obtain IR spectra (recorded in KBr). The structure and basic properties of compounds are presented in Table 1.

**Table 1:** Characteristics of the synthesized iminodiacetic acid derivatives

Compound no.	Molecular structure	Chemical formula	Molecular weight (g/mol)
**1**	$\begin{array}{ll} \text{\emph{N}-(4-methylacetanilide)iminodiacetic acid}\end{array}$	C_13_H_16_N_2_O_5_	280,28
**2**	$\begin{array}{ll} \text{\emph{N}-(2,4-dimethylacetanilide)iminodiacetic acid}\end{array}$	C_14_H_18_N_2_O_5_	294,30
**3**	$\begin{array}{ll} \text{\emph{N}-(2,4,6-trimethylacetanilide)iminodiacetic acid}\end{array}$	C_15_H_20_N_2_O_5_	308,33
**4**	$\begin{array}{ll} \text{\emph{N}-(3-bromo-2,4,6-trimethylacetanilide)iminodiacetic acid}\end{array}$	C_15_H_19_BrN_2_O_5_	387,23

### Aqueous solubility studies

The influence of PAMAM dendrimers on aqueous solubility of the four derivatives of iminodiacetic acid was determined with the equilibrium solubility method. Diluted solutions of PAMAM dendrimers (generation 1–4) in concentration from 0 to 10 mg/ml were prepared. The final volume of the test solution was 500 $\upmu $l. The excess of compounds was then added to each of the test solutions and the obtained suspensions were subjected to ultrasonic effects. The solutions were mechanically shaken for 24 h at 25°C, and then the solutions were centrifuged at 15,000 rpm for 20 min. The saturated solutions were diluted to a proper concentration with double-distilled water, followed by spectrophotometric measurements of absorbance using a Perkin-Elmer UV-Vis spectrophotometer.

Calibration curves were obtained by dissolving a certain amount of every drug in a 0.1:1 (v/v) methanol/water mixture. It was checked that the presence of methanol did not modify the absorbance and the specific wavelength of each drug. The solubility of iminodiacetic acid derivatives in the presence of dendrimers can be calculated according to the measured absorbance and the calibration curve.

Double-distilled water was also used as a blank. Three repeats of each sample were conducted. Furthermore, UV-Vis spectra (range of 190–400 nm) of each sample were prepared.

### Preparation of PAMAM dendrimer—iminodiacetic acid derivative complexes and NMR studies

Complexes of PAMAM dendrimers generation 1–4 with four iminodiacetic acid derivatives were obtained by adding a certain excess of drug to the aqueous solution of PAMAM dendrimer (generation 1–4). Reactions were conducted with 10, 20, 40, and 80 molar excess of compounds 1–4 for complexes with PAMAM dendrimers generation 1–4, respectively. In Table 2, molar ratios for all complexes are presented.

**Table 2:** Molar ratios for complexes between compounds 1–4 and PAMAM dendrimers. Generation 1 and 2

Complex	Substrates (number of moles)	
	Compounds 1–4	Dendrimer
Compound 1–4 with PAMAM dendrimer generation 1.0	10	1
Compound 1–4 with PAMAM dendrimer generation 2.0	20	1
Compound 1–4 with PAMAM dendrimer generation 3.0	40	1
Compound 1–4 with PAMAM dendrimer generation 4.0	80	1

The reaction mixture was stirred for 24 h at room temperature, and then dried under vacuum in order to remove water. The residue was dissolved in deionized water (1 ml) and the solution was centrifuged by means of centrifugal filter devices with 3000 NMWL Ultracel YM membranes (Centricon, Millipore) until the equilibrium point was reached. Then, the residue was dried under vacuum. In this way, we obtained 16 complexes, four for every PAMAM dendrimer generation.

NMR spectra were recorded on Bruker Avance III 600 MHz for ^1^H; the spectrometer was equipped with a standard NMR TBI probe for ^1^H and 2D experiments. The samples were prepared in DMSO-d^6^. All spectra were recorded at 25°C with temperature stabilization. Spectra were calibrated on “rest” DMSO signal at 2.50 ppm.

^1^H NMR spectra of dendrimers, drugs, and mixtures of drugs and dendrimers were obtained. Furthermore, ^13^C, ^1^H-^1^H COSY, ^1^H-^13^C HSQC, and ^1^H-^13^C HMBC spectra were prepared so as to assign properly all signals of drugs and dendrimers.

^1^H-^1^H NOESY spectra were recorded without gradients using standard mixing time 300 ms and 9.95 $\upmu $s ^1^H 90° pulse width. The experiments were done with a 2.03-s relaxation delay and 133-ms acquisition time. Sixteen transients were averaged for each 256 × 2048 complex t_1_ increments. The data were processed with Lorentz-to-Gauss window function and zero filling in both dimensions to display data on a 8192 × 8192 2D-matrix. All data were processed with Bruker Topspin NMR software. All experiments were carried out at 300.0 K with high level of stabilization.

## Results and discussion

### Solubility studies

Solubility enhancement of newly developed drugs has always been a challenge to scientists because the hydrophobicity of these compounds contributes to difficulties during product development and unsatisfactory bioavailability.

Utilization of dendrimers as solubility enhancers has been studied extensively during the last decade. There have been numerous studies that evaluated the effect of various types of dendrimers on solubility of drugs such as nifedipine [29], salicylic acid [30], indomethacin [31, 32], paclitaxel [33], methotrexate [34], flurbiprofen [35], diclofenac, mefenamic acid [36], piroxicam [37], naproxen, ibuprofen, ketoprofen, diflunisal [38, 39], phenylbutazone [40], and nicotinic acid [41].

The use of dendrimers as solubilizing agents has attracted the attention of many scientists due to their characteristic properties, which are different from those of conventional polymers. It has been proven that various types of dendrimers in both original and modified forms contribute to the enhancement of solubility of hydrophobes and that generation number, pH of the solvent, temperature, and dendritic architecture are the factors that influence the efficiency of dendrimers as solubilizing agents. There are a number of studies investigating the effect of generation, pH, and concentration on solubility enhancement; however, the effect of core and temperature remains uninvestigated. It can be concluded that ionic interaction, hydrogen bonding, and hydrophobic interactions contribute to the solubility enhancement.

**Fig. 1:**
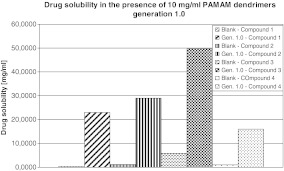
Drug solubility in the presence of 10 mg/ml PAMAM dendrimers, generation 1.0

The effect of G1 PAMAM dendrimer concentration on solubility of four analogues of iminodiacetic acid was measured at 25°C by means of UV-Vis spectroscopy, and the results are presented in Fig. 1. It might be observed that the extremely low water solubility of compound **1** has been significantly improved by G1 PAMAM dendrimers (a 75-fold increase in solubility in 10 mg/ml PAMAM dendrimer solutions compared with that in double-distilled water). Dendrimers contributed to 29-, 10-, and 16-fold increases in solubility of compounds **2**, **3**, and **4**, respectively. However, on the basis of the result of this study, we cannot confirm that PAMAM 1.0 dendrimers contribute to greater solubility enhancement of those compounds which are less soluble in water than those better soluble.

Most researchers have observed that the solubility of a tested drug increased with higher generation number. For example, Cheng and Xu reported that solubility of four NSAIDs (naproxen, ketoprofen, ibuprofen, and diflunisal) was higher in the presence of G4 PAMAM dendrimers than in the presence of dendrimer generation 2 and 3 [38]. On the basis of published studies, there is a tendency to select dendrimers up to generation 4 because they are less immunogenic than dendrimers of higher generations. Experiments evaluating the influence of dendrimers of five and higher generations on the solubility of the drug molecules are described relatively rarely. One example might be an article where researchers estimated that G6 PAMAM dendrimer generation contributed to higher solubility of phenylbutazone [40].

**Fig. 2:**
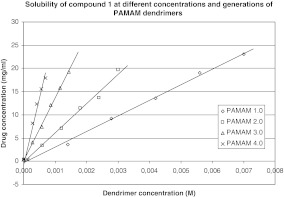
Solubility of compound 1 at different concentrations and generations of PAMAM dendrimers

**Fig. 3:**
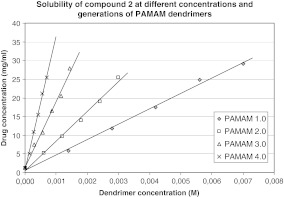
Solubility of compound 2 at different concentrations and generations of PAMAM dendrimers

**Fig. 4:**
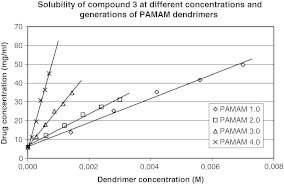
Solubility of compound 3 at different concentrations and generations of PAMAM dendrimers

**Fig. 5:**
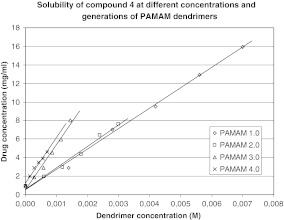
Solubility of compound 4 at different concentrations and generations of PAMAM dendrimers

In our study, the effect of G2–G4 PAMAM dendrimers on the process of solubilization was also investigated. Obtained results are shown in Figs. 2, 3, 4, and 5, from which it is clear that the solubility of iminodiacetic acid derivatives was affected by concentration and generation of PAMAM dendrimers. The solubility of all compounds was the highest in the presence of G4 PAMAM dendrimers, presumably due to the fact that the number of primary and tertiary amines in the dendrimer increases with generation size. Thus, a dendrimer of a higher generation has a tendency to interact with more particles of a hydrophobic compound more than do lower generation dendrimers. Tables S1–S4 (see supporting information) present the mean concentrations of drugs **1**–**4** in the presence of PAMAM dendrimers as well as standard deviations (SD) for all the data.

It is shown that the solubility of all compounds increased significantly with PAMAM dendrimer concentrations. As shown in Figs. 2–5, the apparent solubility of all derivatives of iminodiacetic acid increased in an approximately linear manner as a function of PAMAM dendrimer solution over the whole concentration range. These results might be related to the increase in the number of surface amines and internal cavities that might interact with iminodiacetic acid derivatives. On the surface of PAMAM dendrimers, there are large numbers of primary amines that interact electrostatically with the carboxyl groups of the tested compounds.

The significant solubility enhancement of various drug molecules in the presence of PAMAM dendrimers might be assigned to: (i) the nonpolar cavities in the interior of dendrimers that can entrap hydrophobic drugs by hydrophobic interactions [29, 42, 43]; (ii) cationic functional groups on the surface of dendrimers that can interact with negatively charged drugs by electrostatic interactions [35, 44, 45]; (iii) nitrogen and oxygen atoms in interior cavities of dendrimers that can interact with guests by hydrogen bond interactions [29, 46].

Furthermore, Cheng and coworkers made an attempt to evaluate the role of external electrostatic interaction and internal encapsulation between dendrimers and negatively charged drugs in solubility enhancement of the drugs. They reported that solubility of phenobarbital and sulfamethoxazole increased with the help of PAMAM dendrimers; however, solubility of trimethoprim and primidone (drugs that do not have negatively charged groups) did not increase. Thus, it might be concluded that electrostatic interaction between the negatively charged drugs and dendrimers contributes more to the solubility enhancement than internal encapsulation [47].

In another study, Cheng et al. investigated generation-dependent encapsulation or electrostatic attachment of phenobarbital by PAMAM dendrimers. Dendrimers of lower generation were found to increase the solubility of the drug to a higher degree than higher ones at a fixed mass concentration (2 mg/ml), which is characterized by a similar number of primary and ternary amine groups. It was suggested that solubility enhancement of the drug is caused by electrostatic interactions (the negatively charged form of phenobarbital can be attached to the positively charged PAMAM dendrimers) and internal encapsulation. It was also concluded that dendrimers of lower generation were much more susceptible to electrostatic interaction with a negatively charged drug than the higher generation at a fixed mass concentration. This fact might be explained by much more congested primary amine groups on the surface of dendrimers of a higher generation [48].

Since the tested compounds are weakly acidic, we performed all studies at constant pH conditions (pH = 7). In neutral conditions, these compounds are in ionized form and freely interact electrostatically with the surface amine groups of dendrimers. At this pH, most of the surface amine groups of PAMAM dendrimers are protonated because the reported p*K*a values of the primary amines are 7.0–9.0 [49].

It was reported that solubility of weakly acidic nicotinic acid was the highest at pH 7, and the lowest at pH 3. This is probably due to the fact that at low pH, nicotinic acid exists in unionized form and that the tertiary amine groups are protonated and, thus, the polarity of the environment inside the dendrimer is increased [41]. Furthermore, in the case of furosemide, it was estimated that better results were obtained at pH 4.0–6.0 than at pH = 2. Solubility enhancement was assigned to the electrostatic interaction between the furosemide carboxylic group and the positively charged tertiary amines of the dendrimers [50]. However, Beezer et al. observed that drug–dendrimer complexes were unstable at a pH less than pH 7. It was shown that tertiary amine groups within the dendrimer are important for the binding of guest molecules. Because the p*K*a of tertiary amines in aqueous solution of PAMAM dendrimers is 9.5, tertiary nitrogens are capable of deprotonating the acidic guest molecules and, as a result, create a drug–dendrimer complex. When these groups are protonated (pH = 6 and lower), dendrimers do not possess the ability to bind their guest [30].

### NMR studies

#### ^1^H NMR

^1^H NMR spectroscopy is a powerful, widely used tool that gives information on the presence and types of intermolecular and intramolecular interactions in a host-guest system such as interaction mechanisms, binding sites, and binding affinities [12, 51].

This technique is based on the fact that each nucleus has a unique chemical shift in the NMR spectrum because of its distinct electronic magnetic field. The usefulness of ^1^H NMR stems from the fact that the change in the electronic environment around the target nucleus induces a shield/deshield effect for the nucleus. The downfield or upfield shift of a proton is consistent with the decrease or increase in the electronic cloud intensity around the proton. The decrease of electron density around a nucleus leads to the increase of chemical shift (high-frequency shift), and, vice versa, the increase of electron density around the nucleus causes the decrease of chemical shift (low-frequency shift). Such changes indicate the presence of intermolecular interactions around related protons such as formation of novel inclusion or ion pairs. ^1^H NMR has been extensively employed to investigate the molecular interactions and host-guest chemistry between dendrimers and various drug molecules [52, 53].

The chemical shift assignment of each proton of compounds **1**–**4**, dendrimer generations 1–4, and dendrimer–drug complexes is critical because the shift of each signal is helpful to define the zone of interaction between the dendrimers and iminodiacetic acid derivatives. A specific or nonspecific recognition between a host and a guest can be detected by analyzing the chemical shift variation of a signal from the guest before and after mixing them together.

In order to detect the type of interaction between drugs and dendrimers, ^1^H NMR spectroscopy was applied. The experiments were performed in anhydrous DMSO and were carried out on free dendrimers, compounds **1**–**4**, and their complexes. The encapsulation of guest molecules (compounds **1**–**4**) into the dendrimer structure causes the displacement of the chemical shift of guest protons. This might be evidence of inclusion [45].

The ^1^H NMR spectrum of compound **2 **in DMSO shows several kinds of protons: a broad signal of carboxyl groups (*δ*H 12.55 ppm, 2H, 2xCOOH), a singlet of amide group (*δ*H 9.68 ppm, 1H, NH), two doublets and one singlet coming from protons in aromatic ring (*δ*H 7.71 ppm, Ar-1H, *δ*H 7.02 ppm, Ar-1H, *δ*H 6.97 ppm, Ar-1H), two singlets of methylene protons (*δ*H 3.55 ppm, 4H, 2xCH_2_ and *δ*H 3.42 ppm, 2H, CH_2_) and two singlets of methyl group in an aromatic ring (*δ*H 2.23 ppm, and *δ*H 2.20 ppm, 6H, 2xCH_3_).

Having assigned the chemical shifts of compound **2** in DMSO, we analyzed the complexation of the dendrimer and compound **2** by ^1^H NMR. Chemical shifts of compound **2** in complex with G2 PAMAM dendrimer do not differ significantly from the free drug. However, in complex, there is no signal of the carboxyl groups, which gives evidence of electrostatic interaction between carboxyl groups of the drug and amine groups of dendrimer. Furthermore, a slight upfield shift (from 3.55 to 3.47 ppm) of the methylene protons (4H, 2xCH_2_) adjacent to the carboxyl groups of compound **2** is observed. This slight upfield shift is caused by increased electron density around atoms adjacent to carboxyl groups as a consequence of electrostatic interactions with amine groups.

The same alterations of chemical shifts are observed for complexes between compound **2** and PAMAM dendrimers G1, G3, and G4.

**Scheme 2:**
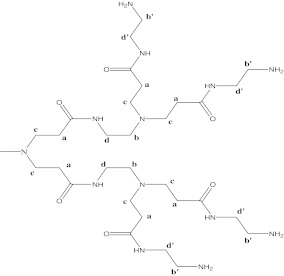
Chemical structure and atom labeling in the PAMAM dendrimers

In the ^1^H NMR spectrum of a G2 PAMAM dendrimer, there are six kinds of ^1^H peaks in DMSO, corresponding to the four CH_2_ protons in the interior of the dendrimer (protons a, b, c, and d; *δ*H 2.21 ppm for a, *δ*H 2.45 ppm for b, *δ*H 2.65 ppm for c, and *δ*H 3.07 ppm for d) and two groups of CH_2_ protons in the outermost layer (*δ*H 2.56 ppm for b′ and *δ*H 3.07 ppm for d′; Scheme 2). Spectra of G1, G3, G4 PAMAM dendrimers present similar chemical shifts for all CH_2_ groups.

**Fig. 6:**
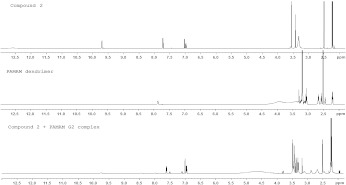
1H NMR spectra of compound 2, G2 PAMAM dendrimer and the complex of compound 2 with G2 PAMAM dendrimer

In complexes between compound **2** and the G2 PAMAM dendrimer, significant changes in chemical shifts of CH_2_ protons (b′ and d′) of the G2 dendrimer are observed. These downfield changes of chemical shift of methylene protons are localized at the outermost layer of the G2 dendrimer (from 2.56 to 2.86 ppm and from 3.07 to 3.29 ppm, respectively) and provide evidence of ionic interactions between terminal protonated amine groups of the dendrimer and carboxylic acid groups of compound **1**. This may be explained by the fact that electron density around the cation (protonated amine group) decreases. The interior methylene protons a, b, c, and d also exhibit downfield shifts. However, this type of change should not be assigned to the electrostatic interaction because these protons are too far away from the surface of the dendrimer. It seems to be due to the quaternization of amine groups in the interior of the G2 dendrimer. This observation suggests that the internal electrostatic interactions may also contribute to the encapsulation of guests with low p*K*a values into the dendrimer structure. Figure 6 presents ^1^H NMR spectra of compound **2**, PAMAM dendrimer G2 and the complex of compound **2** with PAMAM G2 dendrimer.

Similar changes in chemical shifts in methylene protons of dendrimers are also observed in the spectra of complexes between compound **2** and G1, G3, and G4 PAMAM dendrimers.

In the case of drugs **2**, **3**, and **4** and their complexes with PAMAM dendrimers of 1–4 generation similar changes in chemical shifts of methylene protons of the drugs and methylene protons of dendrimers are observed as well, suggesting that both electrostatic interaction between terminal amine groups of PAMAM dendrimers and carboxylic groups of iminodiacetic acid analogues and internal encapsulation of these drugs into the dendrimer structure are the mechanisms of complex formation.

In case of another anionic drug, mycophenolic acid, significant changes in chemical shifts of methylene protons (b′ and d′) of the G5 PAMAM dendrimer were also observed. The downfield chemical shift of these methylene protons localized at the outermost layer of the G5 PAMAM dendrimer is evidence of ionic interactions between primary protonated amine groups of the dendrimer and the deprotonated carboxylic acid group of mycophenolic acid. In contrast to our studies, the interior methylene protons (a–d) of the G5 dendrimer in complex with mycophenolic acid exhibit an upfield shift. It was demonstrated that the distinct shift behavior of protons b′ and d′ and protons a–d in complexes should be assigned to different interaction mechanisms between PAMAM dendrimer and mycophenolic acid. Apart from electrostatic interactions, PAMAM dendrimers may react with mycophenolic acid through hydrophobic interactions and, thus, encapsulate the model drug into the nonpolar interior pockets of dendrimer. Furthermore, there are large numbers of amide groups in PAMAM dendrimers that can act as hydrogen-bond donors in these interior pockets. Therefore, the upfield shift of interior methylene groups (a–d) of the dendrimer might be due to encapsulation of mycophenolic acid in the interior of the dendrimer by hydrophobic interactions or hydrogen–bond interactions [12].

Interactions between G5 PAMAM dendrimers and phenylbutazone were also investigated. In this study, significant downfield shifts of methylene protons b′ and protons d′ in the outermost layer of the G5 dendrimer were observed when phenylbutazone was titrated into the G5 dendrimer solution.

In comparison to mycophenolic acid, many more phenylbutazone molecules were incorporated in the interior dendrimers’ pockets, while more mycophenolic acid molecules were bound on the surface of the dendrimer by electrostatic interactions. This observation might be explained by the fact that phenylbutazone with two aromatic rings and an aliphatic chain is more hydrophobic than mycophenolic acid and that interaction between the carboxylic group of mycophenolic acid with the amine groups of the dendrimer is stronger than the interaction of the carbonyl group of phenylbutazone with the amine groups of the dendrimer [52].

In another study, Cheng et al. [47] evaluated the mechanism of interaction between PAMAM dendrimers and negatively charged drugs: phenobarbital, primidone, sulfamethoxazole, and trimetoprim. In case of phenobarbital, significant shifts of the drug in the presence of G3 or G6 PAMAM were not observed. Thus, it was concluded that the hydrophobic encapsulation is not a predominant interaction type between dendrimers and phenobarbital and that the electrostatic interactions between cationic PAMAM dendrimers and negatively charged drugs are the major force driving the formation of dendrimer–drug complexes and contribute more to the solubility enhancement of hydrophobic drugs than interior encapsulations by hydrogen–bond interactions and hydrophobic interactions. However, it was reported that primidone, despite the fact that it shows similar molecular size to phenobarbital, and similar hydrophobicity, does not interact with PAMAM dendrimers. This is due to the fact that phenobarbital with a low p*K*a value of 7.4 has a weak binding ability with the G5 dendrimer, whereas primidone with a p*K*a value around 13 is not soluble in cationic dendrimer solutions, suggesting no ionic binding between the cationic dendrimer and primidone molecules. This is because phenobarbital generates a negatively charged form in dendrimer solution with a pH value around 10, but primidone is in a noncharged form.

To the best of our knowledge, methotrexate is the only example of a drug containing two carboxylic groups whose interactions with dendrimers have been investigated. Solubility of this drug in the presence of PAMAM dendrimers was not, surprisingly, increased. It was found that two carboxylic acid groups of methotrexate may form cross-linking structures and large aggregates with the multivalent surface of the dendrimers, which form solid precipitates in aqueous solutions during complex formation. This explains well why amine-terminated dendrimers are not able to solubilize the drug molecules [53]. In 2012, Fang et al. evaluated interactions between drugs bearing multiple charges, such as Congo Red and indocyanine green, and PAMAM dendrimers. The authors reported that drug molecules bearing two negative charges form precipitates with cationic PAMAM dendrimers, which lead to complications during the preparation of dendrimer inclusions of these drugs. Fang et al. confirmed by means of NMR techniques that utilization of acetylated PAMAM dendrimers made it possible to obtain stable inclusion complexes with the examined drugs [54].

#### 2D-NOESY analysis

2D-NOESY is a technique that provides information on the distance between protons in close spatial proximity within a given molecule, which is also used to detect host-guest interactions in complexes. If the host and guest are bound, they should be in close proximity to each other. NOE cross peaks should be seen in the corresponding spectral region. The absence of a NOE cross-peak in the region can be used to exclude the interaction between related nuclei [52, 53].

This technique uses the NOE interaction of spins for correlation of protons. The NOE intensity of a cross-peak in the NOESY spectrum is proportional to the number of related protons and the distance between them. In the spectrum, a cross-peak is only observed if the distance between the protons is less than 5 Å [55].

If the dendrimer and guest are bound, they should be close to each other, which can be shown with NOE cross-peaks in the NOESY spectrum. On the other hand, the absence of NOE cross-peak means that there is no interaction between host and guest. The presence and absence of crosspeaks and their intensities in the spectrum enable the determination of the structure of a dendrimer–drug complex.

In our study, the 2D-NOESY technique was utilized to give evidence of the localization of guest molecules within the dendrimer. We obtained ^1^H-^1^H NOESY spectra for every compound **1**–**4** and their complexes with PAMAM dendrimers.

**Fig. 7:**
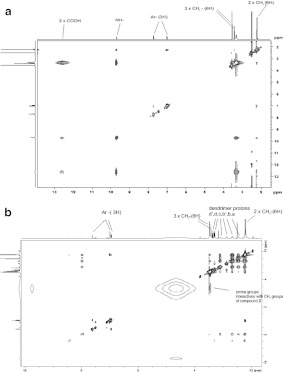
**a** The 1H-1H NOESY spectrum of compound 2. **b** The 1H-1H NOESY spectrum of complex between compound 2 and G2 PAMAM dendrimer

The NOESY spectrum of compound **2** in DMSO solution at a standard mixing time of 300 ms is shown in Fig. 7a. Strong NOE cross-peaks are observed between the carboxyl protons and methylene groups. Weaker interactions are also observed, such as those between the amide group and methylene group, the methyl in the aromatic ring, protons of the aromatic ring, protons of aromatic ring, and carboxyl groups. NOESY spectra of free compounds were prepared in order to properly assign NOE cross-peaks between the drug and dendrimer.

Generally, the mechanism of drug–dendrimer complex formation is based on three types of interactions: (1) external electrostatic interactions between amine groups of the dendrimer and the negatively charged groups of the drug, (2) hydrophobic interactions between the relatively nonpolar pockets of the dendrimer and the drug in the interior of the dendrimer, and (3) hydrogen-bond interactions between ternary amine or amide groups of the dendrimer and functional groups of the drug in the interior of the dendrimer.

In the ^1^H-^1^H NOESY spectrum of the G2 dendrimer-compound **2 **complex in Fig. 6b, there are six ^1^H peaks that correspond to the four CH_2_ protons (a–d) in the interior of the dendrimer and two CH_2_ protons (b′ and d′) in the outermost layer of dendrimer. In the spectrum, strong interactions between the terminal amine groups of the dendrimer and methylene protons of compound **2** are observed.

NOE cross-peaks between protons b′ and d′ of the dendrimers and protons of the guest cannot be observed in Fig. 7b, indicating that compound **2** is not localized in the outermost cavities of the dendrimers. Furthermore, no intermolecular NOE interaction between other protons of compound **2** and the backbone protons of the dendrimer can be found in the spectrum presented in Fig. 7b. This spectrum shows that compound **2** is not localized in the interior cavities, but on the outermost layer of the dendrimer. Similar observations might be made for other complexes of compound **2** and G1, G3, and G4 PAMAM dendrimers.

In case of the previously discussed phenobarbital, 2D-NOESY studies revealed that there are strong interactions between the protons of aromatic ring present in the phenobarbital molecule and those of PAMAM dendrimer of generations 5 and 6. However, no cross-peaks between aromatic protons of phenobarbital and G3 and G4 PAMAM dendrimers were observed, suggesting that few drug molecules were entrapped in the cavities of the G3 or G4 dendrimer. On the basis of 2D-NOESY experiments and solubility studies, Cheng et al. [48] concluded that higher-generation dendrimers are more capable of encapsulating drug molecules into the interior cavities than dendrimers of lower generation. Furthermore, it was stated that dendrimers of lower generation are much better for the electrostatic attachment of phenobarbital molecules than dendrimers of higher generation at a fixed mass concentration [48]. In another study performed by Cheng et al. [47], it occurred that also in the case of sulfamethoxazole, the evidence of encapsulation of this drug was confirmed in a 2D-NOESY experiment for the G6 PAMAM dendrimer, but not for the G3 dendrimer. Thus, on the basis of NMR spectroscopy studies, it may be concluded that the electrostatic interaction contributes more to the solubility enhancement of drugs than encapsulation, especially for low-generation dendrimers [47].

The NOESY spectrum of the G5 PAMAM dendrimer–mycophenolic acid complex has demonstrated the encapsulation of drug molecules in the interior cavities of the dendrimer and the absence of mycophenolic acid molecules localized in the outermost cavities of the dendrimer. The methyl protons of mycophenolic acid showed close proximity with the interior methylene protons a–d of the G5 dendrimer. Furthermore, 2D-NOESY studies revealed that the interactions of the drug with G5 PAMAM dendrimers decrease the spatial distance between the protons of drug molecules. Together with ^1^H NMR spectroscopy, it was confirmed that the mycophenolic acid molecules are bound both on the surface of dendrimers by ionic interactions and in the interior pockets of dendrimers by hydrogen–bond interactions and hydrophobic interactions. The drug-loading efficiency of dendrimers depends on dendrimer concentration, generation, pH conditions, and surface functional groups [12].

Also, in the case of phenylbutazone, the NOESY spectra gave clear evidence that the encapsulation of the drug by the PAMAM dendrimer occurred. NOESY spectra showed the presence of intense cross-peaks between the methyl and phenyl protons of phenylbutazone and the cavity protons of the dendrimers for the G5-phenylbutazone complex [52].

The host-guest interaction is dependent on the deprotonation of the acidic groups in the guest molecule as well as the guest size, hydrophobicity, and the generation of dendrimers. Dendrimers of higher generation are found to be more capable of encapsulating guest molecules in their interior pockets than the lower-generation ones, whereas lower-generation dendrimers are found to be much easier for the electrostatic attachment of guest molecules on the surface than the higher-generation ones. According to the NOESY spectra of all synthesized complexes, where interactions between the terminal amine groups of the dendrimer and methylene protons of compounds were observed, we assume that the examined analogues of iminodiacetic acid were localized on the outermost layer of the dendrimer. On the other hand, no intermolecular NOE interactions between other protons of the compounds and methylene protons of the dendrimer suggest that encapsulation into the interior cavities of PAMAM dendrimers is not a predominant type of interaction between these polymers and iminodiacetic acid derivatives. This assumption might also be explained by the fact that PAMAM dendrimers of lower generations might not be capable of encapsulation of iminodiacetic acid analogues into their interior cavities in large quantities. This statement is similar to the results of the study performed by Cheng et al. [47].

## Conclusions

Herein we described the interaction between PAMAM dendrimers of generation 1–4 and four analogues of iminodiacetic acid. Amine-terminated dendrimers significantly increase the solubility of all synthesized compounds. This enhancement of solubility is linearly connected with concentration of the dendrimer. Furthermore, we reported that the solubility of iminodiacetic acid derivatives in dendrimer solutions likely depends on the dendrimer generation. The order in which the dendrimers increased the solubility of all compounds at a constant pH condition (pH = 7) is G4 > G3 > G2> G1.

To determine the mechanism of interaction between the drugs and PAMAM dendrimers, we applied NMR spectroscopy. ^1^H NMR experiments give evidence that complex formation between the iminodiacetic acid derivatives and PAMAM dendrimers is based on electrostatic interaction between the surface amine groups of PAMAM dendrimer and the carboxyl groups of iminodiacetic acid derivatives. Furthermore, on the basis of alteration of dendrimer methylene protons shifts, we assume that hydrophobic interaction might be the second mechanism of complex formation. 2D-NOESY measurements can provide information on the distance between protons in close spatial proximity within a given molecule and thus can also be utilized to detect the intermolecular interactions between two different molecules. 2D-NOESY studies revealed strong interactions between the terminal amine groups of the dendrimer and methylene protons of iminodiacetic acid derivatives, however, cross-peaks between protons of the iminodiacetic acid derivatives and methylene protons of PAMAM dendrimers were not observed.

The results of our study provide new insight into the host-guest chemistry of complexes between PAMAM dendrimers and drugs containing two carboxylic groups, which might be useful in the design and optimization of dendrimer-based drug delivery systems.

## Electronic Supplementary Material

Below is the link to the electronic supplementary material.

(DOC 137 KB)
