# Supplementary material for: Evaluation of poly(amidoamine) dendrimers as potential carriers of iminodiacetic derivatives using solubility studies and 2D-NOESY NMR spectroscopy
Source: J Biol Phys. 2012 Aug 16;38(4):637–56. doi: 10.1007/s10867-012-9277-5 (PMC3473130; doi:10.1007/s10867-012-9277-5)
Supplement: Supplementary file 1 — (DOC 137 KB) [file 10867_2012_9277_MOESM1_ESM.doc]

| **PAMAM dendrimer concentration (M)** | **Drug's mean concentration (mg/ml)** | **Standard deviation (SD) (mg/ml)** |
| --- | --- | --- |
| **PAMAM 1.0** |  | |
| 0,0000 | 0,314 | 0,0095 |
| 0,0014 | 3,591 | 0,0110 |
| 0,0028 | 9,161 | 0,1080 |
| 0,0042 | 13,612 | 0,1023 |
| 0,0056 | 19,010 | 0,0891 |
| 0,0070 | 23,067 | 0,1182 |
| **PAMAM 2.0** |  | |
| 0,0000 | 0,314 | 0,0095 |
| 0,0006 | 3,425 | 0,0164 |
| 0,0012 | 7,061 | 0,0955 |
| 0,0018 | 11,530 | 0,1192 |
| 0,0024 | 13,688 | 0,1896 |
| 0,0030 | 19,752 | 0,1539 |
| **PAMAM 3.0** |  | |
| 0,0000 | 0,314 | 0,0095 |
| 0,0003 | 4,042 | 0,0410 |
| 0,0006 | 7,438 | 0,1611 |
| 0,0009 | 12,085 | 0,0882 |
| 0,0012 | 15,834 | 0,1197 |
| 0,0014 | 19,295 | 0,1747 |
| **PAMAM 4.0** |  | |
| 0,0000 | 0,314 | 0,0095 |
| 0,0001 | 0,364 | 0,0108 |
| 0,0003 | 8,231 | 0,0894 |
| 0,0004 | 12,350 | 0,1954 |
| 0,0006 | 15,569 | 0,1927 |
| 0,0007 | 17,976 | 0,2051 |

Table S1. Results of solubility studies of compound **1** in the presence of PAMAM dendrimers generation 1 - 4.

| **PAMAM dendrimer concentration (M)** | **Drug's mean concentration (mg/ml)** | **Standard deviation (SD) (mg/ml)** |
| --- | --- | --- |
| **PAMAM 1.0** |  | |
| 0,0000 | 1,156 | 0,0210 |
| 0,0014 | 5,838 | 0,1354 |
| 0,0028 | 11,848 | 0,1085 |
| 0,0042 | 17,522 | 0,1788 |
| 0,0056 | 24,855 | 0,2426 |
| 0,0070 | 29,049 | 0,2405 |
| **PAMAM 2.0** |  | |
| 0,0000 | 1,156 | 0,0210 |
| 0,0006 | 5,192 | 0,1708 |
| 0,0012 | 9,681 | 0,1706 |
| 0,0018 | 13,927 | 0,3540 |
| 0,0024 | 18,934 | 0,2291 |
| 0,0030 | 25,382 | 0,1897 |
| **PAMAM 3.0** |  | |
| 0,0000 | 1,156 | 0,0210 |
| 0,0003 | 7,528 | 0,1655 |
| 0,0006 | 10,764 | 0,1278 |
| 0,0009 | 16,552 | 0,1199 |
| 0,0012 | 20,479 | 0,1174 |
| 0,0014 | 27,898 | 0,1213 |
| **PAMAM 4.0** |  | |
| 0,0000 | 1,156 | 0,0210 |
| 0,0001 | 4,984 | 0,1400 |
| 0,0003 | 10,827 | 0,1321 |
| 0,0004 | 15,544 | 0,1296 |
| 0,0006 | 21,199 | 0,1482 |
| 0,0007 | 25,424 | 0,1900 |

Table S2. Results of solubility studies of compound **2** in the presence of PAMAM dendrimers generation 1 - 4.

| **PAMAM dendrimer concentration (M)** | **Drug's mean concentration (mg/ml)** | **Standard deviation (SD) (mg/ml)** |
| --- | --- | --- |
| **PAMAM 1.0** |  | |
| 0,0000 | 5,829 | 0,0183 |
| 0,0014 | 13,781 | 0,2336 |
| 0,0028 | 25,216 | 0,1953 |
| 0,0042 | 35,204 | 0,1990 |
| 0,0056 | 41,711 | 0,2272 |
| 0,0070 | 49,730 | 0,2560 |
| **PAMAM 2.0** |  | |
| 0,0000 | 5,829 | 0,0183 |
| 0,0006 | 12,001 | 0,1367 |
| 0,0012 | 17,318 | 0,2067 |
| 0,0018 | 23,094 | 0,1954 |
| 0,0024 | 27,240 | 0,2153 |
| 0,0030 | 31,077 | 0,1965 |
| **PAMAM 3.0** |  | |
| 0,0000 | 5,829 | 0,0183 |
| 0,0003 | 11,522 | 0,1496 |
| 0,0006 | 17,887 | 0,1659 |
| 0,0009 | 25,055 | 0,1684 |
| 0,0012 | 28,950 | 0,2108 |
| 0,0014 | 34,860 | 0,1912 |
| **PAMAM 4.0** |  | |
| 0,0000 | 5,829 | 0,0183 |
| 0,0001 | 11,125 | 0,1195 |
| 0,0003 | 19,530 | 0,2108 |
| 0,0004 | 30,813 | 0,2151 |
| 0,0006 | 36,325 | 0,2154 |
| 0,0007 | 45,078 | 0,2022 |

Table S3. Results of solubility studies of compound **3** in the presence of PAMAM dendrimers generation 1 - 4.

| **PAMAM dendrimer concentration (M)** | **Drug's mean concentration (mg/ml)** | **Standard deviation (SD) (mg/ml)** |
| --- | --- | --- |
| **PAMAM 1.0** |  | |
| 0,0000 | 0,972 | 0,0226 |
| 0,0014 | 2,916 | 0,1383 |
| 0,0028 | 7,012 | 0,1630 |
| 0,0042 | 9,526 | 0,1706 |
| 0,0056 | 12,948 | 0,1713 |
| 0,0070 | 15,945 | 0,1978 |
| **PAMAM 2.0** |  | |
| 0,0000 | 0,972 | 0,0226 |
| 0,0006 | 1,931 | 0,0797 |
| 0,0012 | 2,963 | 0,1257 |
| 0,0018 | 4,383 | 0,1413 |
| 0,0024 | 6,462 | 0,2123 |
| 0,0030 | 7,613 | 0,2040 |
| **PAMAM 3.0** |  | |
| 0,0000 | 0,972 | 0,0226 |
| 0,0003 | 1,862 | 0,1326 |
| 0,0006 | 2,874 | 0,1069 |
| 0,0009 | 4,522 | 0,1596 |
| 0,0012 | 5,975 | 0,1657 |
| 0,0014 | 8,021 | 0,2499 |
| **PAMAM 4.0** |  | |
| 0,0000 | 0,972 | 0,0226 |
| 0,0001 | 2,023 | 0,1781 |
| 0,0003 | 2,895 | 0,1589 |
| 0,0004 | 3,465 | 0,2145 |
| 0,0006 | 3,874 | 0,1939 |
| 0,0007 | 4,603 | 0,2283 |

Table S4. Results of solubility studies of compound **4** in the presence of PAMAM dendrimers generation 1 - 4.
